# Assessing the significance of Palaeolithic engraved cortexes. A case study from the Mousterian site of Kiik-Koba, Crimea

**DOI:** 10.1371/journal.pone.0195049

**Published:** 2018-05-02

**Authors:** Ana Majkić, Francesco d’Errico, Vadim Stepanchuk

**Affiliations:** 1 Centre National de la Recherche Scientifique, UMR 5199—PACEA, Université de Bordeaux, Allée Geoffroy Saint Hilaire, Pessac, France; 2 SFF Centre for Early Sapiens Behaviour (SapienCE), University of Bergen, Postboks, Bergen, Norway; 3 Institute of archaeology of NASU, Ukraine; Institucio Catalana de Recerca i Estudis Avancats, SPAIN

## Abstract

Twenty-Seven Lower and Middle Paleolithic sites from Europe and the Middle East are reported in the literature to have yielded incised stones. At eleven of these sites incisions are present on flint cortexes. Even when it is possible to demonstrate that the engravings are ancient and human made, it is often difficult to distinguish incisions resulting from functional activities such as butchery or use as a cutting board, from those produced deliberately, and even more difficult to identify the scope of the latter. In this paper we present results of the analysis of an engraved cortical flint flake found at Kiik-Koba, a key Mousterian site from Crimea, and create an interpretative framework to guide the interpretation of incised cortexes. The frame of inference that we propose allows for a reasoned evaluation of the actions playing a role in the marking process and aims at narrowing down the interpretation of the evidence. The object comes from layer IV, the same layer in which a Neanderthal child burial was unearthed, which contains a para-Micoquian industry of Kiik-Koba type dated to between c.35 and 37 cal kyr BP. The microscopic analysis and 3D reconstruction of the grooves on the cortex of this small flint flake, demonstrate that the incisions represent a deliberate engraving made by a skilled craftsman, probably with two different points. The lines are nearly perfectly framed into the cortex, testifying of well controlled motions. This is especially the case considering the small size of the object, which makes this a difficult task. The production of the engraving required excellent neuromotor and volitional control, which implies focused attention. Evaluation of the Kiik-Koba evidence in the light of the proposed interpretative framework supports the view that the engraving was made with a representational intent.

## Introduction

The emergence of human cognition is a complex issue that needs to be addressed by taking into account diverse and independent lines of evidence. The archaeological record has played a primary role in shaping models accounting for the origin of characteristically human cultures. Perhaps the best known of these models was the one postulating that modern cultures appeared suddenly c. 40 ka in Western Europe [1–5]. However, a string of discoveries made within the last two decades, primarily in Africa, disputed this model and provided the basis for new explanatory paradigms. Some of those tied the emergence of modern cultures and behavior exclusively to the evolutionary history of our own species [6–8], while others envisioned multiple, asynchronous origins among different fossil members of our genus [9–15]. The debate is still ongoing, and especially vivid with respect to attempts to evaluate Neanderthals’ cognitive abilities. A number of interpretative possibilities is offered, ranging from those advocating different [16–29], to those proposing comparable cognition to that of modern humans [9,11,30–39]. The latter view is backed up by new discoveries and reappraisal of old finds. Multiple lines of evidence testify of the innovativeness of Neanderthal cultures, including living space arrangement [40–42], diversified hunting strategies [41,43–46], exploitation of a wide array of resources [47–51], and complex technologies [45,52–55]. However, a recent study has showed that more caution is necessary when drawing inferences on resources exploitation and technological complexity through residue analysis on stone tools [56], challenging some of the previous results (e.g.[49,52]). It is suggested that the latter may require re-evaluation due to possibility of incidental deposition, pinpointing to the inherent difficulty in linking residues on artifacts, especially if not sufficiently abundant, with various food processing activities and/or as evidence for hafting. Neanderthal engagement in symbolic activities is supported by burials [33,57–59], personal ornaments, pigments and pigment-processing tools [35,60–65], collection of exotic objects [64,66], use of feathers and bird bones [67–73], as well as notched, perforated, decorated, and engraved objects or rock surfaces [74–76]. Each of these actions or items entails specific analytical and interpretive challenges.

Abstract engravings, for example, have been reported from numerous Lower and Middle Palaeolithic sites of Europe and Asia (for an overview of the evidence reported in the literature see Table 1 in [77]). Subsequent analyses have shown that some purported engraved designs were better interpreted as natural in origin or resulting from taphonomic processes or post-excavation damage [78,79]. Even when it is possible to demonstrate that engravings are ancient and anthropogenically produced, their interpretation is not a straightforward, unambiguous process as it may be difficult to distinguish incisions resulting from various functional activities from those produced deliberately, and even more difficult to identify the scope of the latter. Microscopic and morphometric analysis of experimental incisions [80–84] provide criteria to infer actions and, to some extent, the intent, behind the production of prehistoric marks (e.g. type of tool used, number of tools involved, direction and chronology of motions, engraver’s laterality etc.). Although results of this type of analysis allow engravings to be formally described, measured, compared, and can guide the interpretation of Palaeolithic markings, they rarely permit a firm identification of their aim and function. A case in point, which is the main focus of the present paper, is represented by engraved cortexes. Twenty-Seven Lower and Middle Paleolithic sites from Europe and the Middle East are reported in the literature to have yielded incised stone objects (Table 1). At eleven of these sites incisions are present on flint cortexes. The largest and one of the best documented collections of the Middle Paleolithic engraved cortexes, including seven specimens, comes from Fumane cave, North of Italy [85]. Found in layers dated to the MIS3-5, these cortexes are associated to both Levallois and Quina technologies. The detailed analysis of these artefacts, and three additional cortexes from the Mousterian layers of San Bernardino Cave and Tagliente shelter, Italy, led the authors of the study to distinguish 1) linear incisions, often present on flat surfaces, interpreted as resulting from cutting activities, 2) sub-parallel incisions produced by scraping to possibly a) clean the flint nodules; b) partially remove the cortex of flakes to better retouch their edges; c) strengthen the hafting of the tool. The approach followed in this study could be complemented in two respects–by considering a wider array of possible reasons for marking cortexes, and by creating an interpretive framework designed to test contrasting hypotheses.

**Table 1:** Contextual and descriptive data on lower and middle Paleolithic stone surfaces with incisions from Europe and Middle East.

Site	Geographic region		Blank		Cultural Attribution	Age (kyr BP)	Total number of objects	Reference
		Lithics	Slab	Pebble				
Axlor	Biscay, SWE	-	-	1	MP	47.5	1	[86]
Baume Bonne	Verdon Gorge, WE	-	-	1	LP	MIS6,5	1	[87]
Bilzingsleben	Thuringia, CE	-	1	-	LP	370	1	[88]
Brno-Bohunice	The South Moravian Region, CE	-	-	1	MP-B	42	1	[87,89]
El Castillo	Cantabria, SWE	-	1	-	MP-LM	40–38	1	[90,91]
Champlost Cave	Yonne, WE	1	-	-	MP-M	MIS3	1	[92]
Chez Pourré-Chez Comte	Correze, WE	-	-	4	MP-M	MIS3	4	[92]
Combe Grenal	Dordogne, WE	-	-	>1	MP-M	MIS 5,4	>1	[87]
Erd	Pest County, CE	-	-	1	MP-M	45–35	1	[93,94]
Grotta dell'Alto	Apulia, SE	-	-	1	MP-M	MIS3	1	[95]
Grotta del Cavallo	Apulia, SE	1	1	1	MP-M	MIS3	3	[96]
Grotta di Fumane	Veneto, SE	7	-	2	MP-M	MIS5,4,3	9	[85,97]
Grotta Maggiore di San Bernardino	Veneto, SE	2	-	-	MP-M	59–44	2	[85]
Grotte de l'Observatoire	Monaco, WE	1	-	-	LP-A	MIS6	1	[87,98]
Grotte du Loup	Correze, WE	-	1	-	MP-C	40–35	1	[87,99]
Hermies	Somme, WE	1	-	-	MP-M	MIS5a	1	[87,100]
Isturitz	Pyrenees-Atlantiques, WE	1	-	-	MP-M	MIS4,3	1	[87,101]
Kiik-Koba	Crimea, EE	1	-	-	MP-PM	32	1	[102]
Lazaret	Mount Boron, WE	-	-	18	LP-UA	MIS6	18	[87,103]
Markkleeberg	Saxony, CE	-	-	1	LP	c.250	1	[87]
Riparo Tagliente	Veneto, SE	3	-	1	MP-M	MIS3	4	[85,97,104,105]
Temnata Dupka	Balkans, SEE	-	1	-	MP	50	1	[106,107]
Terra Amata	Mount Boron, WE	-	-	3	LP	380	3	[87,108,109]
Tsonskaia	Caucasus, EE	-	1	-	MP-LM	MIS 3	1	[110,111]
Qafzeh	Golan heights, ME	1	-	-	MP-M	100	1	[112]
Quneitra	Golan heights, ME	1	-	-	MP-M	50	1	[113,114]
Mar-Tarik	Kermanshah, WA	-	1	-	MP-M	123	1	[115]

A: Acheulean; B: Bohunician; C: Chatelperronian; CE: Central Europe; EE: Eastern Europe; LM: Late Mousterian; LP: Lower Paleolithic; M: Mousterian; ME: Middle East; MP: Middle Paleolithic; PM: para-Micoquian; SE: Southern Europe; SEE: South-Eastern Europe; UA: Upper Acheulean; WA: Western Asia; WE: Western Europe.

Frames of inference have been developed to analyse Palaeolithic markings on other raw materials. The study of utensils used in different human cultures worldwide to store nonlinguistic information has identified for example clues to recognize such implements in the prehistoric record [116,117]. The degree of intentionality reflected by sequential notches cut on a Mousterian bird bone has been recently evaluated by comparing the coefficient of variation of selected metrical variables, calculated on the archaeological specimen, with that measured on sequences of notches made by modern experimenters under similar neuromotor constraints [73]. Results suggest that the Neanderthal craftsman incised the bird bone with the intention of producing equidistant notches, implying that Neanderthals were perceiving and discriminating equidistant from unequally spaced sequential marks in a manner similar to modern humans, and that their neuromotor control allowed them to master the techniques and motions necessary to obtain regularity when required. The combined application of the last two theoretical approaches to Early Later Stone Age and Mousterian objects bearing sequential notches has shown that devices to store numerical information were used in Africa before the European Upper Palaeolithic, and that they may have also been used by Neanderthals well before the arrival of modern humans in Europe [118].

In this paper, we present the results of the analysis of an engraved cortical flint flake found at Kiik-Koba, Crimea, well known for being the first East European Mousterian site that has yielded Neanderthal remains, and create a framework to guide the interpretation of incised cortexes.

### A frame of inference for interpreting engraved cortexes

Incisions can be made on cortical areas of flint nodules, cores or flakes of different size, surface flatness, provenance, quality, and color, bearing cortexes of different texture and thickness, with tools made of different material (wood, bone, antler, stone), different morphologies of the tool’s active area (pointed, irregular, linear), different motions (single or back-and-forth movement), directions relative to one another (unique, multiple, random) or relative to the object or cortical area shape (parallel, oblique, perpendicular to the cortex or the object`s main axis), executed with different degrees of neuromotor control, of varied length, depth, and angle, covering surfaces of different size and at different locations relative to the cortical area or the object outline (central, eccentric, pervasive), during single or multiple sessions, and with the same or different tools. Consideration of these features combined with a technological analysis of the incisions [75,80,83] and their chronology with respect to phases of debitage and retouch can help to identify the course of action to which nodules and cortical flakes were submitted, and possibly identify the underlying logic and intentions behind those actions.

The reasons for producing incisions on a cortex can be multiple. They can be produced before or after knapping, accidentally or intentionally, result from varied utilitarian and/or non-utilitarian activities. Incisions can be produced accidentally on a cortex while, for example, extracting the flint nodule from its primary or secondary deposit. They can be made in order to: clean the nodule by removing attached sediment or the cortex’s more powdery layer, test the properties of the raw material (e.g. evaluate the thickness of the cortex), collect the resulting calcareous powder, use the nodule as a hammer to knapp other nodules or to retouch flakes, facilitate prehension during the knapping/retouching of other nodules/flakes or of the nodule itself, reduce the thickness of the cortex to knapp the nodule in a more effective way, use the nodule as a cutting board or as a grinder, indicate to a novice where to strike, communicate iconic, indexical or symbolic meanings such as the source where the nodule was collected, the owner(s) of the nodule etc., result from an activity such as doodling, represent markings testifying of counting, numbering, or playing. Similarly, incisions can be produced on a cortical flake after it has been detached from the nodule in order to: facilitate prehension during the use of the flake, recover the calcareous powder, remove the powder released by the cortex to prevent its incorporation in the material processed by the flake (meat, skin, vegetal matter etc.), strengthen the hafting of the tool by improving adherence of an adhesive or a twine, thin the cortex to better retouch the flake`s edge, use the flake as a retoucher, use the flake as a cutting board, communicate a variety of iconic, indexical or symbolic information concerning, for example, the owner(s) of the flake, the task it should be used for, recording of the various information, or result from activities such as doodling or playing. A number of reasons listed above for incising the cortex of nodules and flakes are also applicable to cores.

**Fig 1:**
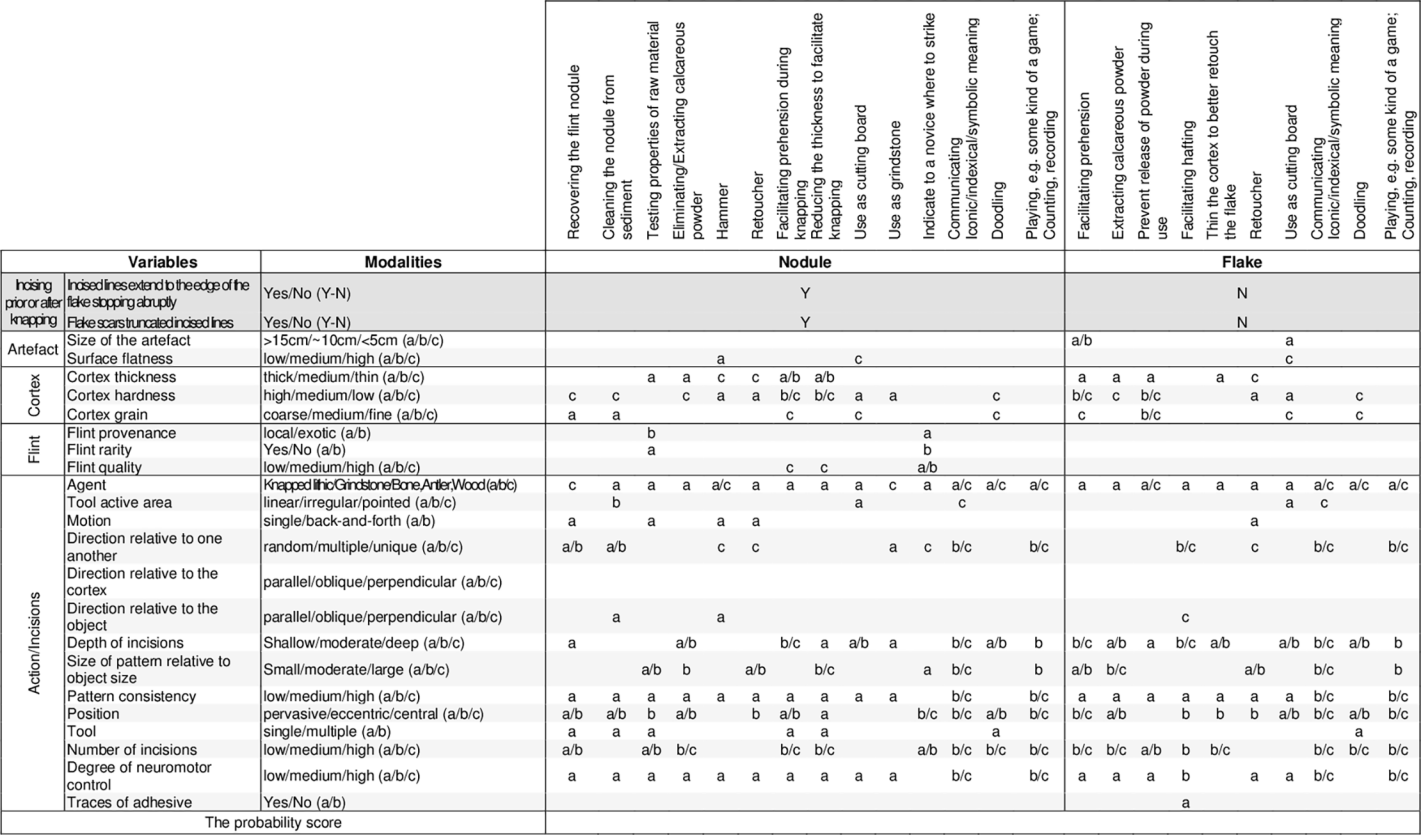
A framework for assessing the character of the engravings on cortexes.

Some technical choices, actions, or suites of actions producing markings on cortexes are incompatible with some reasons listed above for producing incisions; other are fully consistent with one or more reasons. For example, scraping a flint nodule with the unretouched edge of a flake, which results in patches of indistinct superficial parallel striations, better matches the hypothesis that the nodule was cleaned to remove sediment and cortex powder rather than it was engraved with the aim of recording a symbolic information. Incisions partially removed by flake or retouch scars indicate that they were produced before those actions took place. We propose here the frame of inference as a methodological aid for organizing and considering in a synthetic manner the above listed possible reasons for incising cortexes, allowing a reasoned evaluation of the actions playing a role in the marking process. The proposed framework, aimed to narrow down the interpretation of the evidence, systematizes qualitative and quantitative variables relevant for assessing the character of the incisions in a form of a cross-check table (Fig 1) in which material, actions, and outcome are evaluated with respect to the possible purposes of those actions. Modalities relative to the former are made explicit to facilitate use of the interpretive framework by other researchers and are employed to predict the degree of intentionality reflected by the analysed artefacts. The list of variables includes 24 items characterizing the object and the incisions. The variables are classified in five categories—one eliminative and four descriptive. The former is aimed to discriminate if the incisions were executed before or after the knapping of the nodule. The four descriptive categories provide information on qualitative and metric data, characteristics of the cortex and the flint material, as well as incisions and actions employed to produce them. Modalities for each variable, indicated by letters, are arranged in a progressive order where applicable. Hypotheses accounting for the possible reasons for engraving the cortex are considered separately for the phases before ("Nodule”) and after knapping (“Flake”). The relevant variables for evaluating the likeliness of each hypotheses are pre-indicated by the expected modality for each case.

The modalities are assigned scores, leveled as following: 0/0.5/1. A score of 1 is assigned for the match between expected and observed characteristic, a score of 0 for the absence of the match, and a score of 0.5 for a partial match. The lack or ambiguity of pertinent information is indicated with an “x”. In such cases, the results for the variables marked with an “x” encompass the entire range of possible scores by providing the minimum and maximum values. The average of the individual results gives a total probability score for each hypothesis. This score ranges between 0 and 1, with 0 indicating the lowest, and 1 the highest likeliness for the tested hypothesis. The score is not seen as an indicator allowing to firmly prove or disprove hypotheses but rather as an aid to guide the interpretation of the artefact.

## Materials and methods

### Archaeological context

**Fig 2:**
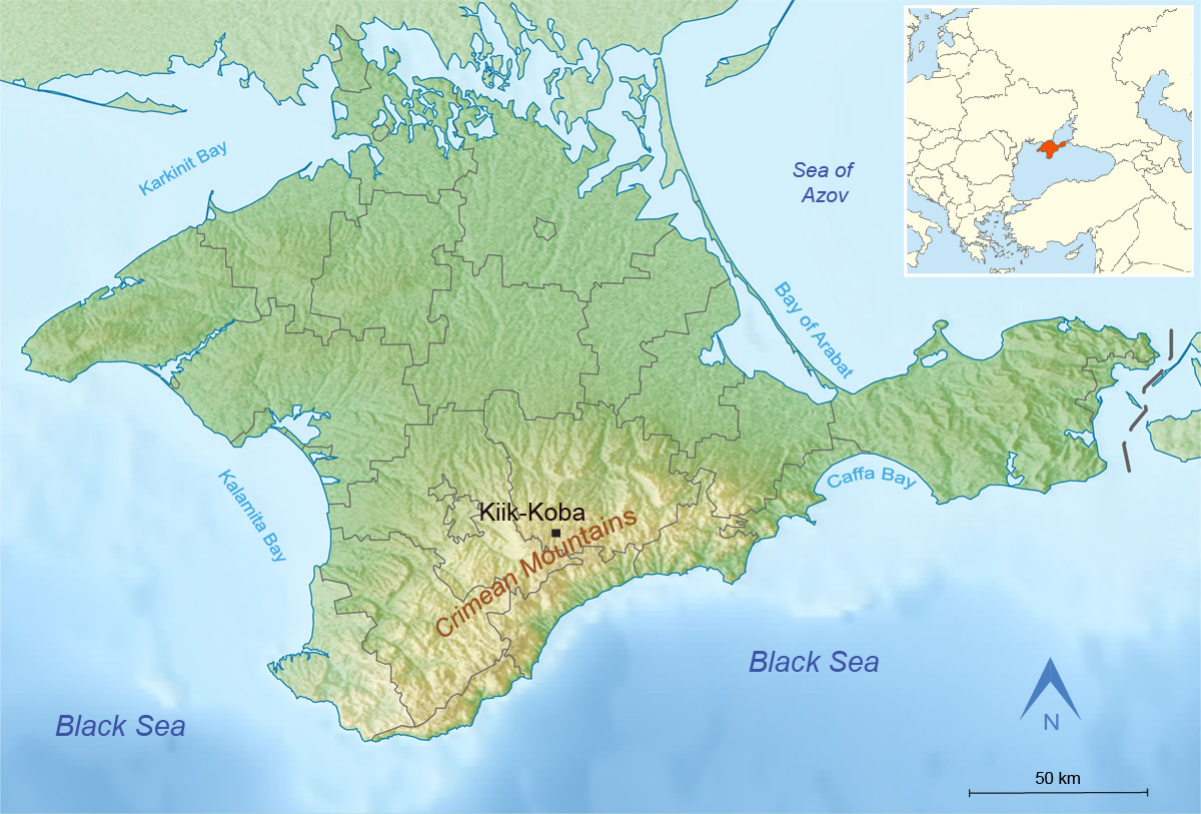
Location of Kiik-Koba cave, Crimea. Modified after Fig 1 in [73].

Kiik-Koba is a large cave site discovered in 1924 by Bonch-Osmolovsky and investigated by him between 1924 and 1926. It is located (44°57' N, 34°21' E) 7 km south of the town Zuya, Belogorsk district, on the right bank of the Zuya river, at an altitude of 512 m above sea level and 90 m above the river bank (Fig 2). Like most stratified Paleolithic sites of Crimea, this cave is formed by fluvial erosion cutting through the second ridge of the Crimean Mountains. Well known for being the first East European site that has yielded Neanderthal remains [119,120], the site has been intensively investigated [119,121–124]. Now the cave contains no sediments, though control areas were left by Bonch-Osmolovsky [125].

**Fig 3:**
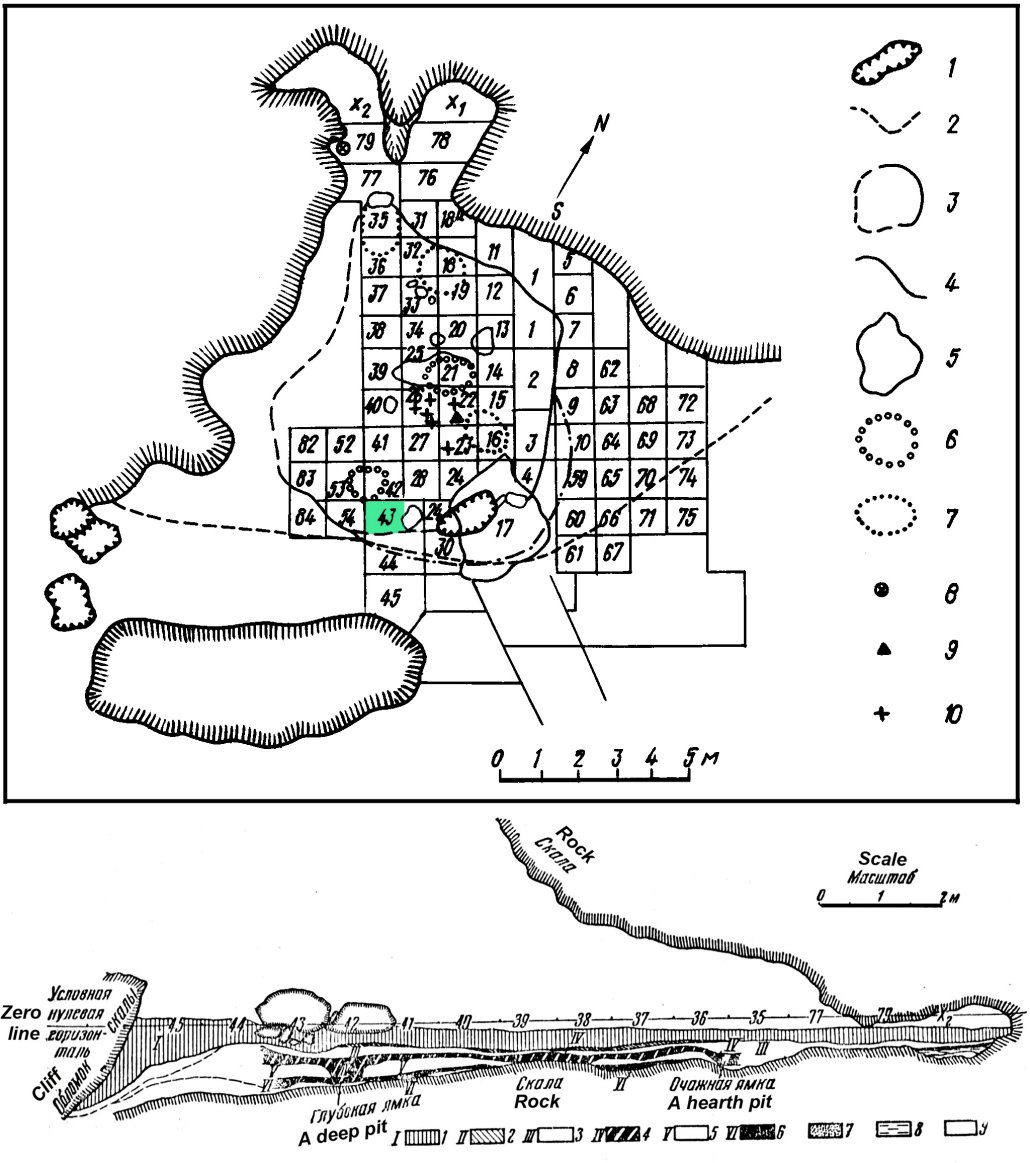
Ground plan and stratigraphy of Kiik-Koba cave. Top: plan of Kiik-Koba cave indicating the excavated area, the extent of the two main Middle Paleolithic occupations and the square meter in which the engraved cortex described in the present study was recovered. Bottom: stratigraphical profile along the south-west limit of the squares 45–35, 77, and 79 X2. Modified after [122].

The sedimentary sequence increases in depth from the back (20–30 cm) to the entrance (1.5 m) of the cave (Fig 3).

Seven main lithologically well defined layers labeled by Bonch-Osmolovsky, from the top to the bottom, I-VII, were interpreted as reflecting two distinct MP occupations, separated by sterile yellow sediments of 10–15 cm thickness [122,125–127]. Layers VI and V, conventionally called layer VI, include remains of the lower MP occupation. Layers IV to II, conventionally referred to as layer IV, are associated with Upper MP occupation, which is characterized by a marked decrease in the number of lithic artifacts and faunal remains. Mesolithic artifacts were found in layer IIa, only identified on an erosional slope at the entrance of the cave. The excavation covered an area of c.150m^2^ and removed almost completely the archaeological layers. The lower occupation extended over a surface of c.70m^2^, the upper occupation of c.50m^2^. The former yielded 12,874 lithics, the latter 4,755. Mesolithic layer IIa contained 50 pieces. Square meter and layer of provenance were recorded for all lithic artifacts and faunal remains. Sediment from archeological layers was systematically sieved. Nearly completely excavated archaeological surfaces of layer IV, also reveal evidence of deep pits dug from layer IV into the underlying layer VI, which resulted in admixture of a part of lithic material [122]. Although it is not easy to estimate the precise extent of potential admixture, the analysis of the site formation process implies that the number of artifacts originally associated with layer IV needs a critical revision as not all of lithics reported for layer IV correlate to human activities in that level [122]:36; [127].

Lithic assemblages from the lower and the upper MP layers significantly differ in technology and typology. The former is defined as a Taubachian [128,129] or as a denticulate MP; the latter, which includes foliate pieces, as a Micoquian [130]. Kiik-Koba layer IV represents the para-Micoquian of Kiik-Koba type, for which the typical “Keilmesser” are not common [102,131], in contrast to Micoquian assemblages of Ak-Kaya type [63]. Typologically, the assemblage was also described as Kiik-Koba industry type of Crimean Micoquian tradition characterized by small dimension of lithics and intensive reworking [132].

Technologically, layer IV industry is characterized by centripetal method of knapping; though atypical Levallois, irregular, and sub-parallel reduction strategies are also present. Bifacial shaping is common. The predominant flake tools are points, followed by sidescrapers, and rare backed knives. Bifacial tools, mostly points, and less frequently sidescrapers and backed knives, account for 14% of the retouched pieces. Three fireplaces and seven pits, containing lithics and bone fragments, were recognized in layer IV. This evidence, as well as refittings of flint artifacts, bones in anatomical connection, difference in preservation of the faunal material between the two layers, and existence of a sterile sediment that prevented significant admixture of archaeological finds support the stratigraphic integrity of the site [122,123]. Evidence for the building of shields protecting the habitation area from south-eastern winds was also published [133], but this hypothesis is challenged due to the lack of sufficient data that would support it [125].

Upper layer (IV) was initially associated with the Mindel-Riss interglacial, the end of Riss-Würm /beginning of Würm or the Brøup interstadial [122,131,134]. The ^14^C dating (32300 ± 300 Ki-8163; 37026–35486 Cal BP (95.4%)—calibrated with OxCal 4.2 online software using the IntCal 13 curve) place this occupation at the end of MIS3 ([102]: 108–109). This ^14^C age is consistent with those obtained at other sites from Crimea, which attribute an age of 35–29 ky BP [130,135,136] to sites with comparable lithic assemblages.

Two Neanderthal skeletons–an adult and an infant–were discovered in the central part of the cave, one meter apart. Identification of artificial pits that, in the case of the adult burial Kiik-Koba 1, apparently involved excavation of the bedrock, support intentional burial. The adult skeleton was significantly damaged by a later pit. The infant skeleton Kiik-Koba 2 was more complete but poorly preserved. The stratigraphical attribution of the possible burials is controversial. The common view is that Kiik-Koba 1 is associated with lower layer VI and Kiik-Koba 2 with layer IV [57,58,125,137].

**Fig 4:**
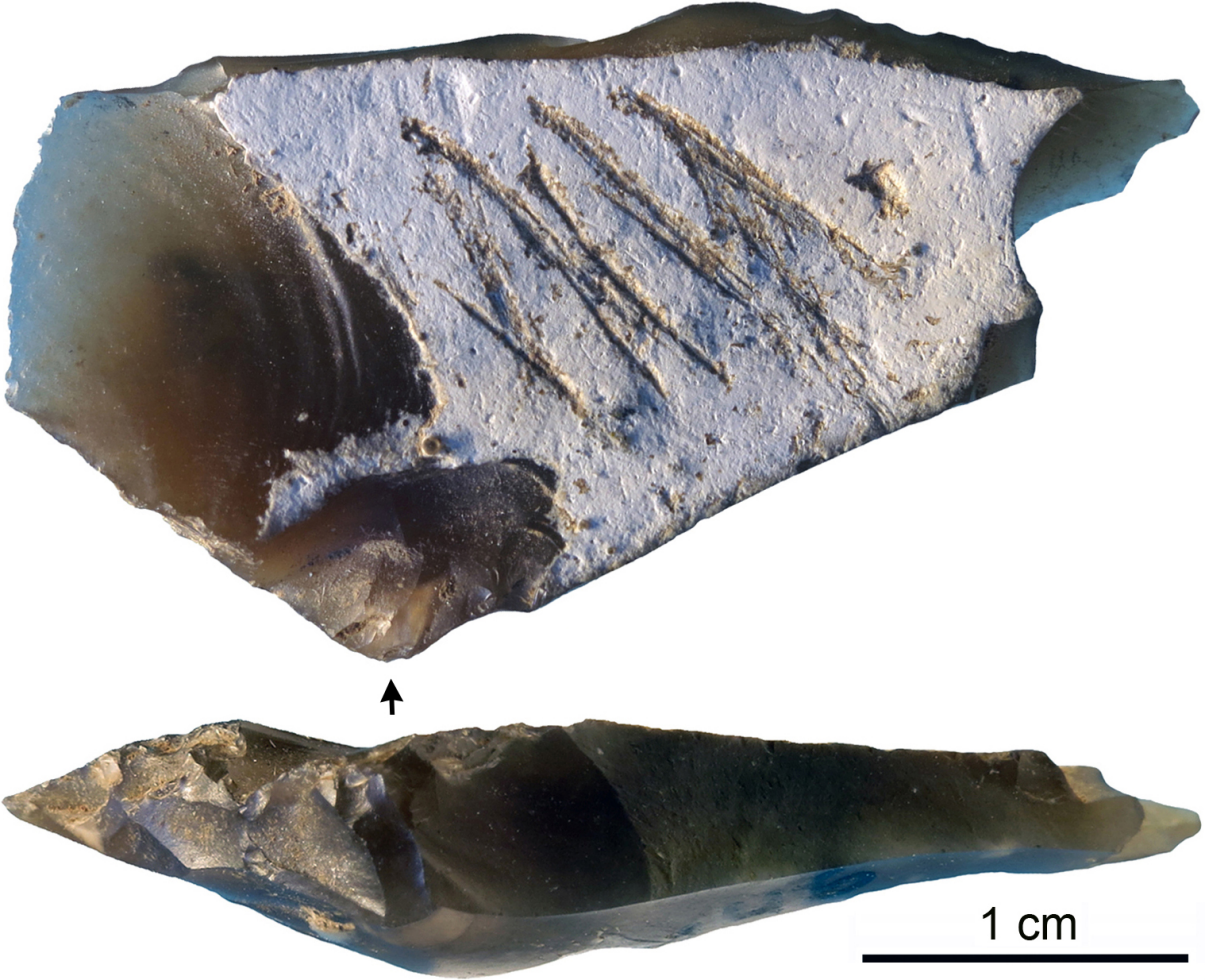
The engraved flint flake from Kiik-Koba layer IV. The arrow indicates the point of impact. Scale = 1 cm.

The artifact analyzed in this paper (Fig 4) comes from a stratigraphically undisturbed square of layer IV and was briefly described by Stepanchuk as an intentionally incised object, possibly reflecting non-utilitarian activities performed by Crimean Neanderthals [102].

### Layer IV

This 10–15 cm thick layer is composed of a dark-brown gravelly loamy soil, rich in decomposed organic material. Lithics are typically made of a fine-grained semitransparent brownish-yellow flint and more rarely, of dark gray, gray, and dark-brown flint [122]. The former is only found in layer IV (Table 2). The majority of artifacts from layer IV are not patinated—only 10% feature a very light patina and some of them bear calcareous dendrites [122].

**Table 2:** Color variations per layer of flint artefacts from Kiik-Koba. After [122]: 99.

Coloring	Layer VI		Layer IV		Layer IIa	
	N	%	N	%	N	%
Dark-gray	291	46	69	14	-	-
Gray yellowish or bluish	187	30	45	9	-	-
Light-gray	72	11	-	-	-	-
Brown	43	7	-	-	-	-
Black	26	4	17	3	-	-
White patinated	3	1	-	-	-	-
Yellow	4	1	-	-	-	-
Brown semitransparent	-	-	331	69	-	-
Dark-brown	-	-	27	5	-	-
Bluish-gray	-	-	-	-	~50	100
TOTAL	626	100	479	100	~50	100

No flint outcrops were found in the vicinity of Kiik-Koba, and the flint from distant outcrops, located near Zuya and Belogorsk towns is different from that found at Kiik-Koba. Currently inaccessible sources may be local, as hypothesized by Bonch-Osmolovsky [122], or remote as argued on the basis of the high degree of exhaustion and intensive reduction of artifacts [127,132,138].

Table 3 describes the main features of the lithic assemblage as given by the author of the excavations and on the basis of a recent reexamination, conducted by one of us (VS) of the material stored in Saint-Petersburg [122,131]. Additional analyses of the assemblage from Kiik-Koba layer IV have been carried out, with recent results published in a monograph devoted specifically to this subject [127,132,138]. The cores are rare and include flat centripetal unifacial (1) and bifacial (6), sub-parallel (1), and atypical Levallois forms (1) [102,131].

**Table 3:** Main typological features of the Kiik-Koba lithic assemblage from layer IV.

	Kiik-Koba, layer IV, after [122]	Kiik-Koba, layer IV, after [131]
Total number of tools	766	615
Among them flake tools	663 (85.4%)	529 (86.0%)
Among them bifacial tools	103 (13.4%)	86 (14.0%)
**Flake tools**		
Total N (= 100%)	663 (= 100%)	529 (= 100%)
Points	270 (40.7)	206 (38.9)
Sidescrapers	330 (49.8)	172 (32.5)
Knives	-	25 (4.7)
Denticulates	-	54 (8.7)
Notched tools	-	8 (1.5)
Endscrapers	-	8 (1.5)
Burins	1 (0.2)	2 (0.4)
Borer (percoirs)	-	1 (0.2)
Piece esquille	-	1 (0.2)
Indetermined fragments	62 (9.4)	114 (21.6)
**Bifacial tools**		
Total N (= 100%)	103 (= 100%)	86 (= 100%)
Handaxes	3 (2.9)	-
Leafpoint	-	1 (1.1)
Points	61 (59.2)	39 (45.4)
Sidescrapers	28 (27.2)	16 (18.6)
Backed knives	-	5 (5.8)
Indetermined fragments	11 (10.7)	24 (27.9)

Small 2–3 cm long flakes predominate. Indices, calculated on 160 tools from a collection No. 5391, stored in the Peter the Great Museum of Anthropology and Ethnography in St. Petersburg, indicate variable preparation of striking zones (IFlarge– 41,9; IFstr– 21,9), which are comparable to those calculated on the basis of the data provided by Bonch-Osmolovsky (IFlarge = 33.5; IFstr = 20) [122]. Blade products are rare among the unretouched pieces (Ilam = 5.4 (by the series of unretouched pieces) and only slightly more frequent among the retouched tools (Ilam = 11.6). Levallois products are absent. Fully cortical flakes account for 3.7% and partially cortical flakes for the 51.6% of the assemblage ([131]: 56).

Points are the most numerous among flake tools (206) with canted forms being predominant (117). Sidescrapers are represented by simple (75), double (31), convergent (17), and canted (48) forms. Backed knives (25), notched and denticulate pieces (62), and other forms are also present, including few endscrapers and a burin. Bifacial forms are represented by a leaf-shaped point (1), points (39), sidescrapers (16), and backed knives (5). The assemblage also includes pebble and flint retouchers [131].

According to Demidenko`s analysis of Kiik-Koba IV lithic assemblage [138], general tool structure was represented through three categories, including: unifacial tools (68.8%), retouched pieces (15.1%), and bifacial tools (16.1%) comprising the total of 417 items. Unifacial tools were represented by scrapers (47.5%), points (34.2%), denticulates (1%), notches (0.7%), and varia. Scrapers and points constitute the basic set of unifacial tools. Scrapers are represented by simple (35.3%), transverse (16.9%), double (8.1%), and convergent (39.7%) types. Among bifacial tools, points are predominant (55.3%), followed by convergent scrapers (36.8%), single-edged scrapers (5.3%), and a denticulate (2.6%). High percentage of fragmented pieces is indicative for intensive exploitation. General composition of the assemblage is associated with a trend toward markedly intensive flint exploitation [132,138].

Uthmeier [127] restricted analysis to artifacts larger than 3 cm, mainly made from brownish flint associated with layer IV, excluding those made from grey to dark grey flint associated with the lower layer, as well as the products of discoidal flaking, also postulated as characteristic exclusively for layer VI. Consequently, the re-analyzed assemblage composed in this manner was smaller, but Uthmeier`s comparison between thus obtained results, with tool frequencies reported by Stepanchuk [131] and Demidenko (Table 9.9 in [139]) combined, converge to the similar conclusion that points, simple side scrapers, and surface shaped (bifacial) tools dominate among formal tool categories ([127]: 143, 156). Most differences in characterization of layer IV lithic assemblage account for the frequencies of formal tools [132], but similar conclusions are reached in respect that unifacial and bifacial points, as well as the small dimensions of formal tools, stand out as the most striking features of layer IV assemblage [127].

The fauna from the upper MP occupation of Kiik-Koba includes giant and red deer, saiga, horse, mammoth, Pleistocene donkey, bison, brown bear, and other species (Table 4). Pollen spectra reflect a steppe environment with a larger representation of trees (birch, pine, oak, hazel) than in the lower layer (M.N. Klapchuk`s in [102]). In addition, juniper, buckthorn, and most likely maple, were identified from the charcoal remains (A.F. Gammerman and I.V. Palibin data in [122]).

**Table 4:** Faunal remains from Kiik-Koba, layer IV.

Species	NISP	MNI	Reference*
*Elephas sp*.	42	2	[122,140]
*Bos sp*.*/ Bison sp*.	12	1	[122,140]
*Ovis sp*.	1	1	[122,140]
*Saiga tatarica*	144	5	[122,140]
*Megaloceros giganteus*	236	8	[122,140]
*Cervus elaphus*	16	1	[122,140]
*Sus scrofa*	2	1	[122,140]
*Rhinoceros antiquitatis*	5	1	[122,140]
*Equus caballus*	103	6	[122,140]
*Equus (asinus) hidruntinus*	3	2	[122,140]
*Canis lupus*	3	1	[122,140]
*Vulpes vulpes*	13	2	[122,140]
*Vulpes lagopus*	12	3?	[122,140]
*Vulpes corsac*	27	5	[122,140]
*Ursus arctors*	2	1	[122,140]
*Hyaena spelaea*	10	1	[122,140]
*Lepus europeus*	1	1	[122,140]
*Marmota bobac*	2	2	[122,140]
*Citellus birulae*	7	5	[122,140]
*Citellus cricetus*	1	1	[122,140]
*Apodemus flavicollis*	1	1	[122,140]
*Lagurus luteus*	2	1	[122,140]
*Alactaga jaculus*	1	1	[122,140]
*Apus apus*	+	-	[122,140]
*Pyrrhocorax pyrrhocorax*	1	1	[122,140]
*Pyrrhocorax graculus*	+	-	[122,140]

* After data provided by [122]:69 and [140].

Faunal remains are heavily fragmented. They often display signs of human modification inflicted during butchering and use of long bone fragments as retouchers [122,141]. Semenov [142] identified traces of cutting and grinding on a horse mandible, interpreted as a mortar. A pointed bone tool was also found.

The object analyzed in this paper comes from the undisturbed part of layer IV, square 43 (Fig 3). It was found in 1925, first identified in 1990, and published in 2006 [102]. According to Bonch-Osmolovsky ([122]: 26), the square 43, located at the very edge of the layer, close to the drop line, has yielded less than 75 lithics. Limits of layers IV and VI were clearly recognizable in this area ([122]: 41) and the presence of intermediate lithological layer V excludes the possibility of mixing between the two main archaeological layers. The stratigraphic sequence of the square 43 had contained layers II to VII, and the former was sealed by a large lime-stone block ([122]: 21, Fig 10), which excludes a possibility of admixture of material from layer IV with that from the Epi-Palaeolithic layer.

### Flake with incised cortex from layer IV

The object analyzed in this study is kept at the Institute of Archaeology of NASU, Kiev, Ukraine. Its catalog number is KkK/IV:006/015.32. Metric data on the archaeological object were acquired with a digital caliper. A NIKON D5300 and a Canon PowerShot S100 digital cameras were used to create the high-quality images of three aspects of the object, and to make macrophotographs of areas of interest. Digital images were edited in the Adobe® Photoshop® CS5.1 Extended software; tracing of the object and the observed modifications was done in the Adobe® Illustrator® CS5.1 software. In order to identify and photographically document natural and anthropogenic modifications, the object was examined with a Leica Z6 APOA motorized microscope equipped with a DFC420 digital camera. The obtained images were treated with Leica Application Suite (LAS) Multifocus module, and Leica Map DCM 3D software. By relying on the adapted algorithms that combine digital images collected at different heights into a single, sharp, composite image, the LAS Multifocus module enables the acquisition of extended depth of field images. The microscopic images were digitized and edited in the Adobe® Photoshop® CS5.1 Extended software. The 3D reconstructions of the areas of interest were produced with the Leica Map DCM 3D software, used also to examine and record the morphometric data on the incisions. Identification of the nature and origin of the modifications recorded on this object is based on the experimental reproduction and microscopic analysis of incisions produced on bone and stone surfaces with different tools and motions [80–83,116].

## Results

### Description and technological analysis

The object analysed in this article (length: 35.86; width: 18.14; thickness: 5.75 mm) is a high-quality flint primary flake detached from a small nodule collected in a primary deposit, as indicated by the good state of preservation of the soft chalky cortex covering most of the dorsal surface (Fig 4). Two tangential negative scars partially removing the cortex on the left side of the flake`s dorsal surface reflect previous attempts to remove the cortex from the nodule without wasting the inner high quality raw material. The small size of the bulb, and the flake thinness confirm the knapper’s intention to produce thin invasive cortical flakes in order to maximize exploitation of the volume of a small nodule. The distal oblique edge corresponds to a plunged termination. The concave fracture on the right side of the flake, and associated microscars visible on the ventral face of the flake in this area, may have resulted from utilization. This is also probably the case for adjacent microscars observed on the right side of the plunged termination and along the left edge. Flakes similar to the one analyzed here are described by Bonch-Osmolovsky [100].

The cortex bears a group of sub-parallel, occasionally overlapping, straight or slightly curved incisions. The presence, at places, of concretion sediment inside the lines, similar staining of black mineral oxides inside and outside the lines, and no detectable change in patina between the two, demonstrate that the lines are ancient and cannot have resulted from excavation or curation damage. Their depth, clean edges, internal parallel striations resulting from protuberances of the marking agent indicate that the lines were produced by one or multiple lithic pointed tools (see below). With a single exception, consisting of very superficial striations that barely mark the cortex, incisions do not reach the cortical surface edge and appear consistently concentrated in the center of it. Their completeness and nearly perfect framing indicate that the lines were incised after the flaking and the simultaneous removal of the distal and right flake fragments took place.

**Fig 5:**
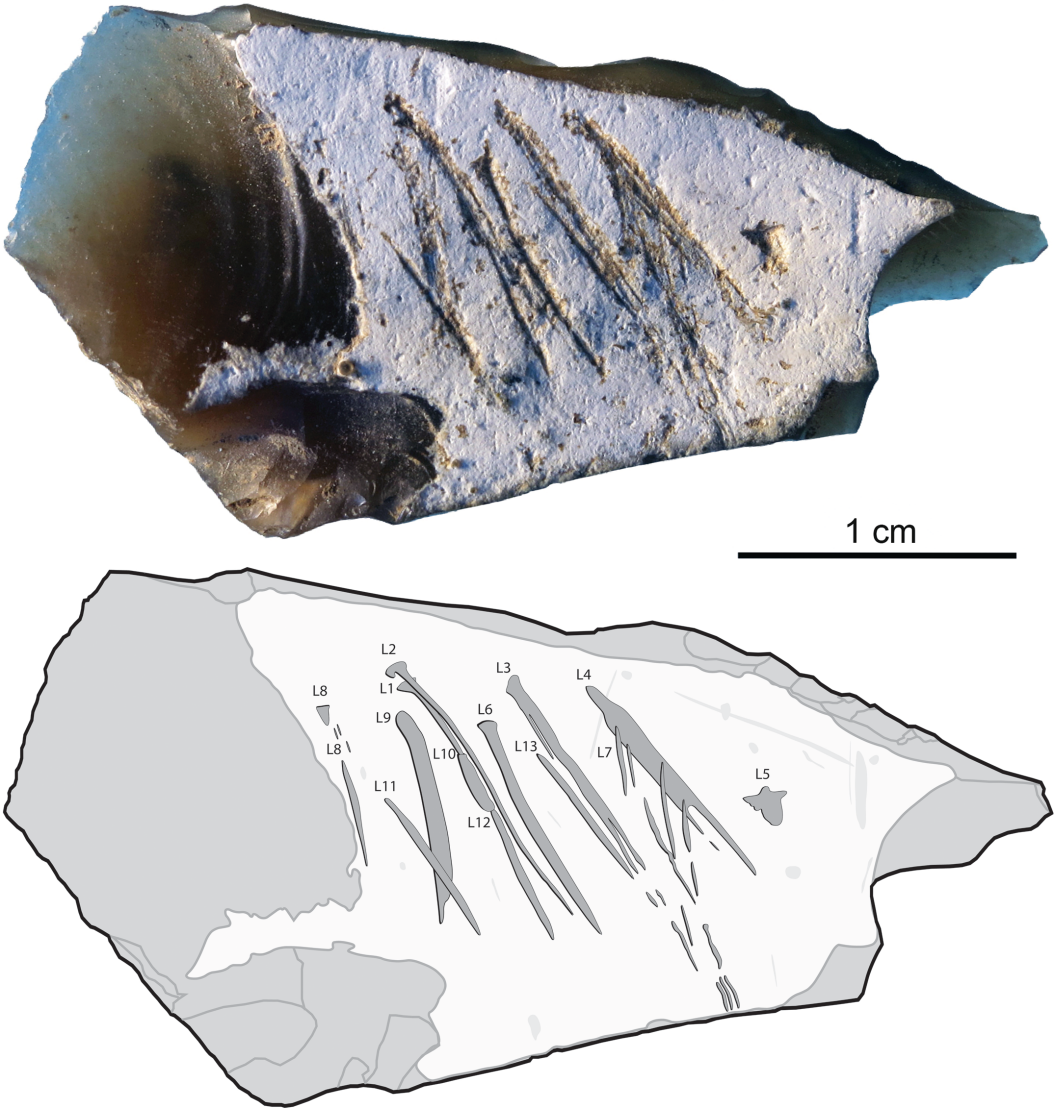
The engraved flint flake from Kiik-Koba, layer IV. Photo (top) and tracing (bottom). Engraved lines are indicated by dark-grey areas outlined in black, surface damage by light gray areas, flake scars by gray lines.

**Fig 6:**
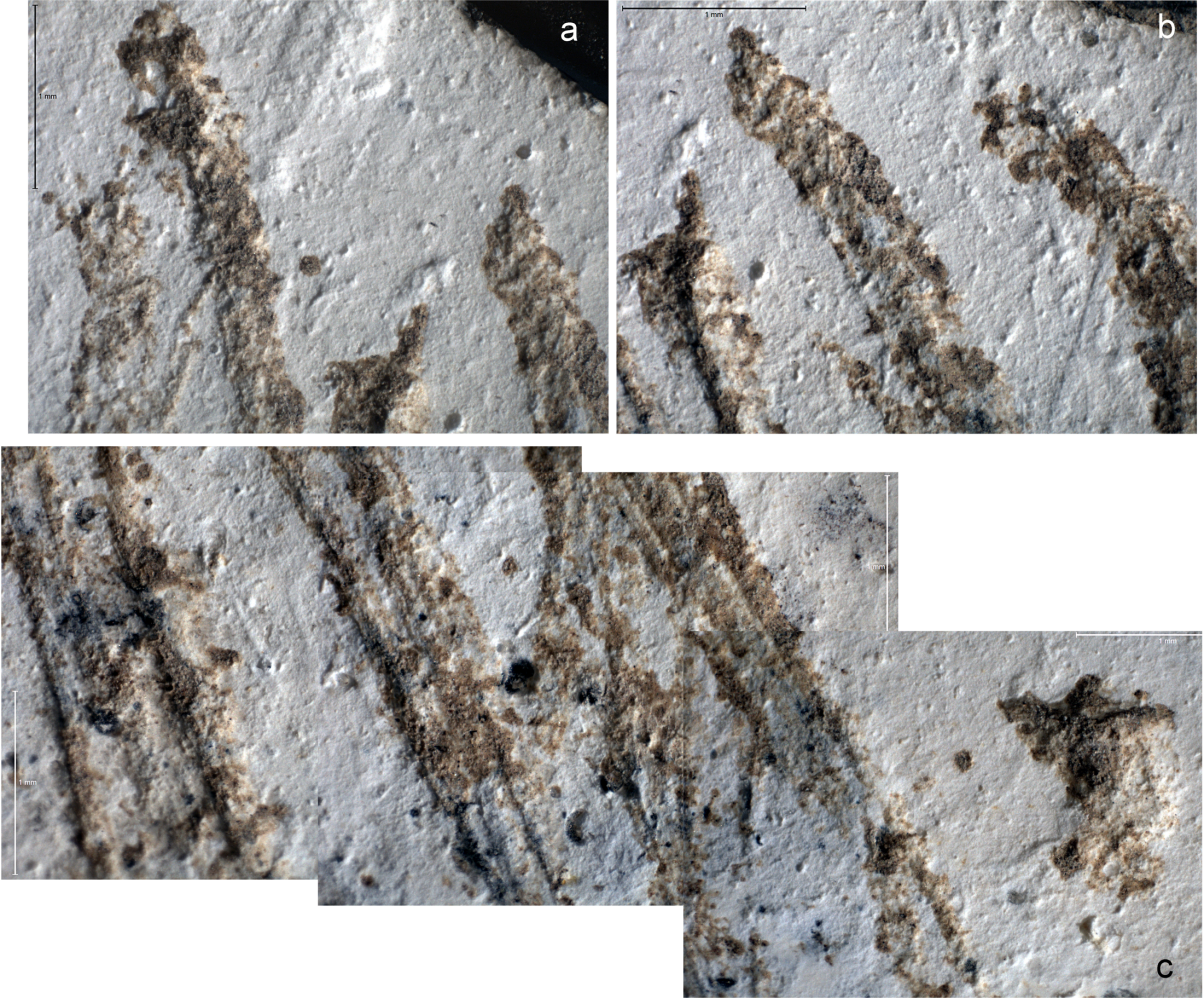
Close-up views of selected lines on the Kiik-Koba engraved cortex. a: from left to right, starting points of lines 9, 1, 2, 6, and 3; b: from left to right, starting points of lines 6, 3 and 4; c: from left to right, middle of the lines 12, 2, 13, 3, 7, and 4. Scales = 1 mm.

Microscopic examination of the incisions`outlines, internal morphology and crossings allow us to propose a technological interpretation of the incised pattern. The lines were produced by moving the engraving tool towards the flake striking platform. This is demonstrated by the well-defined morphology of most lines’ starting points and their systematic fading out at the opposite ends. Thirteen single stroke lines were made on the cortex, called from now on L1-13 (Fig 5 and Fig 6). Three distinct cross-sections and starting points morphologies are identified: 1) a thin v-shaped with a broad triangular well defined and deep starting point (incisions L1-L7), 2) a broad superficial with a flat bottom marked by distinct internal striations and a superficial ill-defined starting point (incisions L8-L10), 3) a thin, deep, slightly asymmetrical to the right with a pointed starting point (incisions L11-L13).

**Fig 7:**
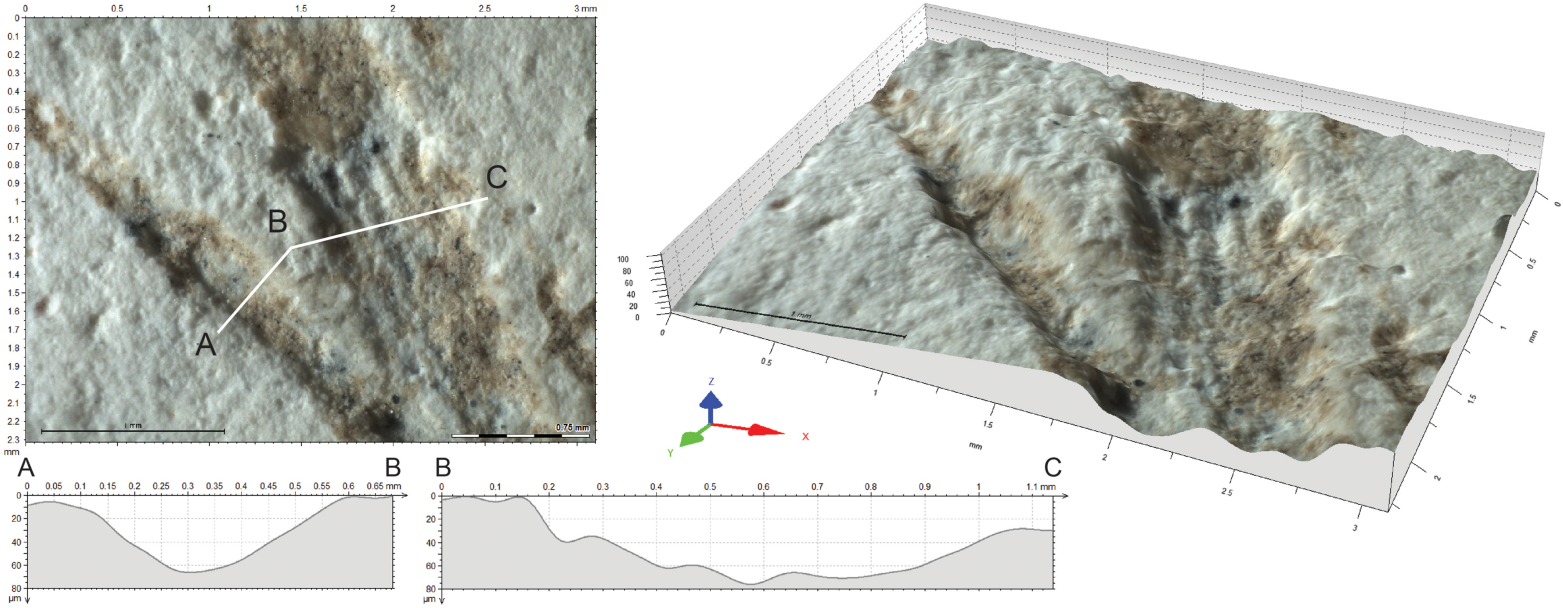
Intersection between L9 and L11 and their cross-section. Top left: intersection between L9 and L11 with location of the sections A-B and B-C; Top right: 3D reconstruction of the intersection; Bottom: sections of the two lines.

The asymmetry of the last type supports the idea that the engraver was a right-handed person. The chronology is established based on one crossing between L9 and L11 (Fig 7), and two overlapping (L1, L10, L12, and L4, L7), the overlapping of L1 and L2 being too filled of concretion sediment to assess the order of these two lines on the basis of microscopic features.

**Fig 8:**
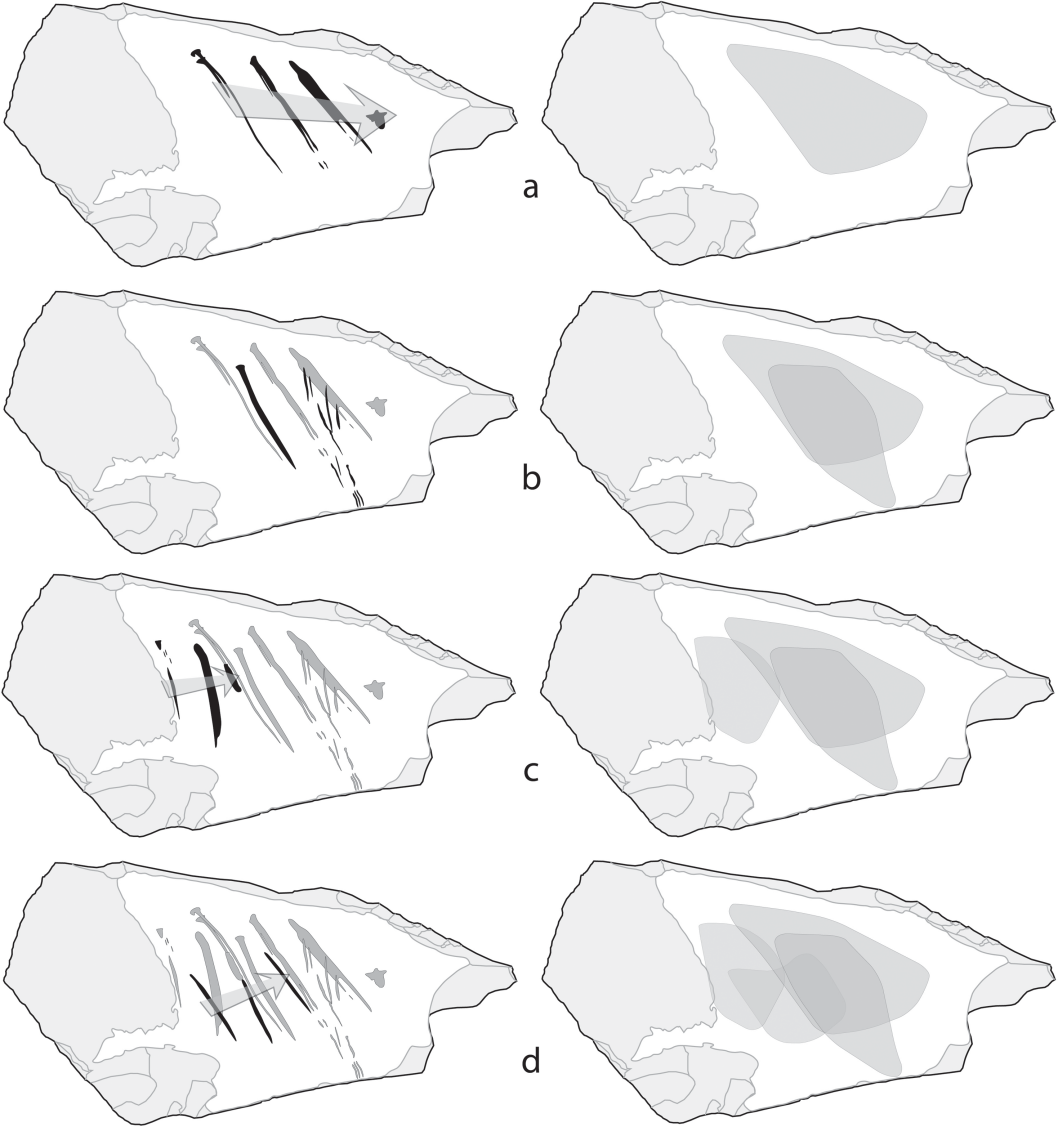
**Probable chronology of the incisions on the Kiik-Koba cortex (left)**. Note the areas of the cortex concerned by each new engraving episode indicating intent to fill in the center of the cortex (right).

The engraving probably started with L1 or L2 (Fig 8A). The shortness of L1 suggests that it was the first incised and that L2 was made immediately afterwards over it to extend L1. The use of the same point, producing a triangular-shaped head, is clearly recognizable on L1, L2, L5, and L6. Similarity in direction and arrangement between L1, L2, L3 and L4 indicate that L2 was probably followed by L3 and L4 in rapid succession, using the same tool, but applying more pressure. L6, which is deeper, differently oriented and features an opposite curvature, may have been added between L2 and L3 at this stage, to fill in the gap between these two lines or, more probably, after L5, a deep puncture produced by exerting a strong pressure on the cortex surface. L7, made after L4, consists of a juxtaposition of superficial parallel striations produced by a single or double passage of a point establishing a discontinuous contact with the cortex surface. The distance between these striations suggests that the tool may have been the one responsible for L1-4. L8 is an incision composed of two isolated segments, a triangular puncture followed, after a gap partially filled with microscopic indentations, by a superficial line with internal striations. Although its superficial nature makes a definite assessment problematic, this line bears a degree of similarity with L9 and L10, equally superficial and displaying a flat bottom covered by striations. The gap and indentations followed by a new line morphology observed on L8 may have resulted from the break of the point used for previous incisions L1-6 as shown by experimental reproduction of single stroke lines on pebbles ([80]: 24–25). This implies that in spite of morphological differences between incisions, the same tool was likely used to produce the first ten lines. L11-13 were the last made (Fig 8) and are shorter, deeper and less parallel than those comprising previous sets and particularly L9 and 10. The engraver may have changed tool, used the same tool but changed its orientation, or used a different protuberance of the same tool in order to avoid producing superficial lines similar to L9 and L10.

Consideration of the lines`chronology and the areas of the cortex concerned by each new phase of the engraving (Fig 8) suggest that the aim of the Neanderthal craftsman was to fill in the center of the cortical surface with sub parallel lines without getting close or intersecting the cortex edge. In order to achieve this goal, the engraver has combined four procedures: 1) juxtaposing lines from left to right, 2) incising lines in the space left between already engraved lines, 3) starting new sets of lines at the left of previously engraved sets, 4) slightly changing the orientation or the curvature of new lines. The overall depth of the lines and the change of point between L10 and L11, executed with the apparent intent of producing deeper lines, are consistent with the idea that the engraver aimed to create a perceptible contrast between the engraved area at the center and the surrounding white cortex. The above is not contradicted by L7 and L8, which reach the edge of the cortex. The morphology of the lines composing the pattern, and in particular their ends, indicate that they were made with quick motions, performed in rapid succession, almost certainly during a single session. L7 and L8 are very superficial and do not alter the visual pattern created by the remainder of the lines. They probably correspond to mistakes in the positioning of the tool due to the speed of execution of the overall pattern.

## Discussion

**Fig 9:**
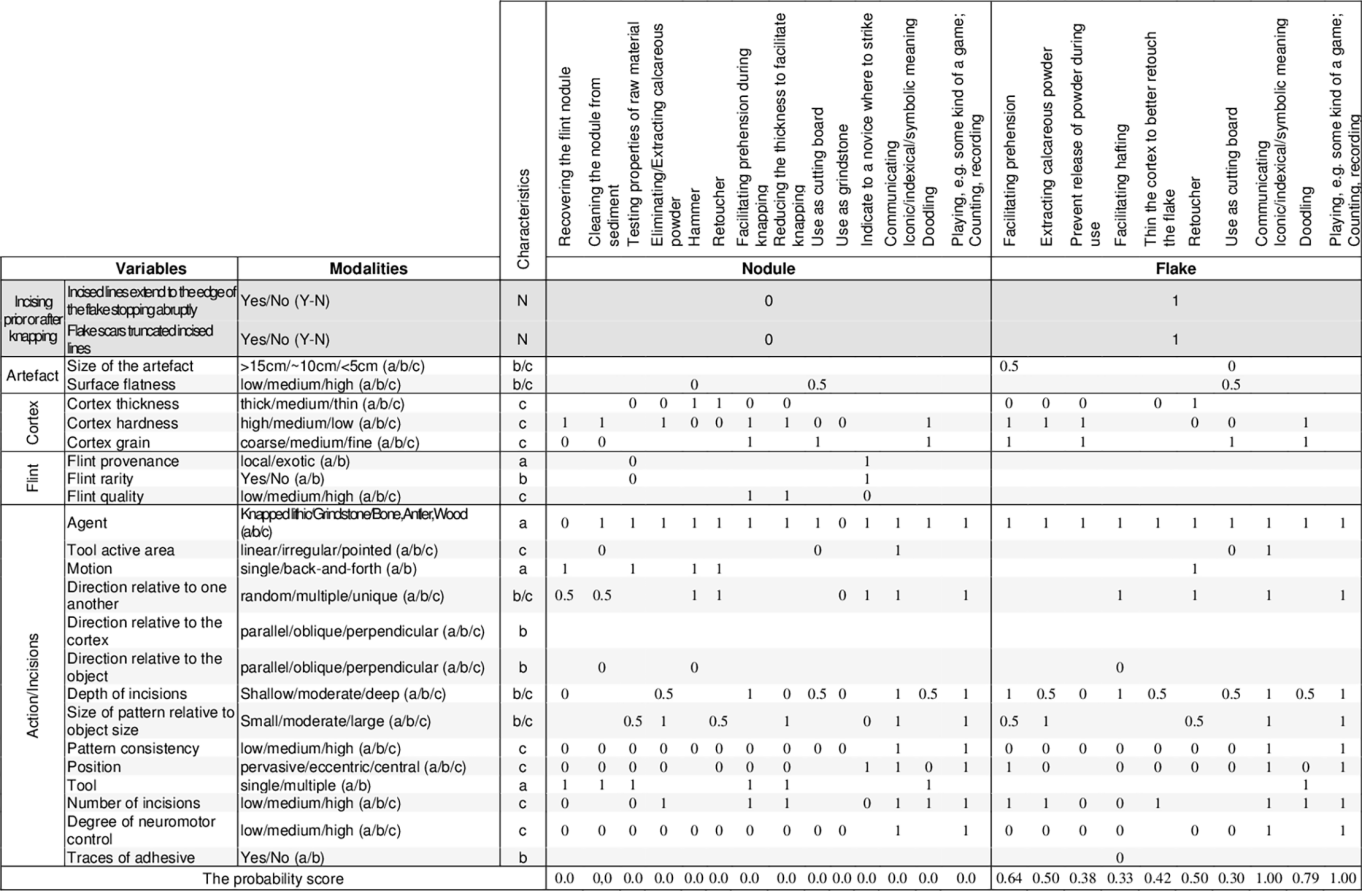
Systematization of the analysis results of the engraved cortex from Kiik-Koba.

Results of the analysis of the Kiik-Koba engraved cortical flake are formalized in the interpretative framework proposed in this study (Fig 9). The latter serves as an effective tool to systematically narrow down and eliminate some hypotheses, and combine all available information in order to evaluate the pertinence of others. The location of the engraving on the cortex and the way in which the lines were produced indicate that the cortex was engraved after the flake was detached from the core, eliminating the causes that may have led to marking the cortex of a nodule. A number of causes for producing markings on a flake can also be discarded. The lines on the cortex are clearly different from the impacts produced when using a soft pebble as a retoucher [143–145]. Although flat, the cortex is too small to have been used as a cutting board, and the lines bear diagnostic features indicating that they were made by a point rather than the edge of knapped stone tool in a cutting motion. The surface of the cortex is too small and the cortex too thin to accept as reasonable the hypothesis that the craftsman’s intention was that of extracting calcareous powder or preventing the release of such powder during the use of the flake. The flake scars present on the dorsal face were either removed before the flake was detached from the nodule or consist of marginal micro-flake scars resulting from the use of the tool. Both are inconsistent with the hypothesis that scraping was applied to increase the invasiveness of a retouch. Increasing the adherence of an adhesive to haft the object, or coating it with an organic compound to facilitate prehension during its use as a tool, is unlikely due to the small size of the object and because the starting points of the lines are very close to the cutting edge, bearing micro-flake scars interpreted as resulting from use. Coating this area with an adhesive would have left a very narrow active zone, thus reducing the effectiveness of the tool`s edge in a scraping or cutting action. Although ameliorating prehension may appear as a more viable possibility, one needs to consider that the surface of the cortex on the flake is smaller than that of a fingertip and that due to its thinness the flake would break easily if used in a task requiring vigorous motions. In addition, this interpretation does not explain why the craftsman has taken care, when engraving each new set, to carefully stop the lines before reaching the edge of the cortex and leave an empty band around the engraved area. Doodling is generally defined as the action of aimlessly producing patterns unrelated to the primary task [146], without focused attention specifically dedicated to the process. Since doodling takes place during a certain lap of time, it usually requires a fairly large surface to be performed, and is also inherently linked to the possibility of marking that surface at ease, relatively effortlessly, while attention is directed elsewhere. The tiny surface of the cortex and the focused attention necessary to engrave the lines with precision counters the doodling hypothesis. The above renders a purely functional or nonpurposive aim for the engraving less likely. Thus, we should consider the possibility that the pattern was deliberately engraved to express and/or communicate a meaning. The discovery of a similar flake, which bears no engraving, in the same layer, indicates that marking good quality cortical flakes was not a systematic behavior at Kiik-Koba. This fact makes it unlikely that the object represented a special case of marking ownership. The fact that the lines are in some cases superimposed and difficult to distinguish visually suggests that a symbolic and counting functions would be unlikely for the pattern taken in isolation, or for its individual elements. Instead of lines taken in isolation, it is rather the contrast between the whitish background and the heavily hashed center of the cortex that may have been used to recall an information to the flake user or eventually communicate one when the tool was passed to somebody else. This fact and the small size of the engraving are consistent with the possibility that the sign was intended to convey an information only to a small number of individuals. The precision with which engraving was executed indicates very good hand-eye coordination and motor skills employed with effort, attention to detail, and an intent to frame the incised pattern within the given cortical surface. This could be consistent with possible representational interpretation of the object.

The interpretative framework proposed here should be extended to include Upper Paleolithic and other Mousterian engraved cortexes in order to tune its heuristic power on a variety of cultural contexts and types of modifications (see S1 and S2 Tables–systematization of the results on the two oldest known engraved cortexes from the Middle East—from the sites of Qafzeh and Quneitra, on the basis of data as reported in dedicated publications [112–114]). For the time being, the results of this study add to the growing body of evidence that Neanderthal cultural adaptations, particularly those at the end of their cultural trajectory, included practices that could be consistent with symbolic interpretations.

## Supporting information

S1 Table Systematization of the results of the engraved cortex from Qafzeh. (DOCX)

S2 Table Systematization of the results of the engraved cortex from Quneitra. (DOCX)
