# Supplementary material for: Assessing the significance of Palaeolithic engraved cortexes. A case study from the Mousterian site of Kiik-Koba, Crimea
Source: PLoS One. 2018 May 2;13(5):e0195049. doi: 10.1371/journal.pone.0195049 (PMC5931501; doi:10.1371/journal.pone.0195049)
Supplement: S1 Table — (DOCX) [file pone.0195049.s001.docx]

**S1 Table. Systematization of the results of the engraved cortex from Qafzeh.**

|  | |  | Characteristics | Recovering the flint nodule | Cleaning the nodule from sediment | Testing properties of raw material | Eliminating/Extracting calcareous powder | Hammer | Retoucher | Facilitating prehension during knapping | Reducing the thickness to facilitate knapping | Use as cutting board | Use as grindstone | Indicate to a novice where to strike | Communicating Iconic/indexical/symbolic meaning | Doodling | Playing, e.g. some kind of a game; Counting, recording | Facilitating prehension | Extracting calcareous powder | Prevent release of powder during use | Facilitating hafting | Thin the cortex to better retouch the flake | Retoucher | Use as cutting board | Communicating Iconic/indexical/symbolic meaning | Doodling | Playing, e.g. some kind of a game; Counting, recording |
| --- | --- | --- | --- | --- | --- | --- | --- | --- | --- | --- | --- | --- | --- | --- | --- | --- | --- | --- | --- | --- | --- | --- | --- | --- | --- | --- | --- |
|  | **Variables** | **Modalities** |  | **Nodule** | | | | | | | | | | | | | | **Flake** | | | | | | | | | |
| Incising prior or after knapping | Incised lines extend to the edge of the flake stopping abruptly | Yes/No (Y-N) | Y | 1 | | | | | | | | | | | | | | 0 | | | | | | | | | |
|  | Flake scars truncated incised lines | Yes/No (Y-N) | Y | 1 | | | | | | | | | | | | | | 0 | | | | | | | | | |
| Artefact | Size of the artefact | >15cm/~10cm/<5cm (a/b/c) | b/c |  |  |  |  |  |  |  |  |  |  |  |  |  |  | 0.5 |  |  |  |  |  | 0 |  |  |  |
|  | Surface flatness | low/medium/high (a/b/c) | c |  |  |  |  | 0 |  |  |  | 1 |  |  |  |  |  |  |  |  |  |  |  | 1 |  |  |  |
| Cortex | Cortex thickness | thick/medium/thin (a/b/c) | a |  |  | 1 | 1 | 0 | 0 | 1 | 1 |  |  |  |  |  |  | 1 | 1 | 1 |  | 1 | 0 |  |  |  |  |
|  | Cortex hardness | high/medium/low (a/b/c) | b | 0 | 0 |  | 0 | 0 | 0 | 1 | 1 | 0 | 0 |  |  | 0 |  | 1 | 0 | 1 |  |  | 0 | 0 |  | 0 |  |
|  | Cortex grain | coarse/medium/fine (a/b/c) | c | 0 | 0 |  |  |  |  | 1 |  | 1 |  |  |  | 1 |  | 1 |  | 1 |  |  |  | 1 |  | 1 |  |
| Flint | Flint provenance | local/exotic (a/b) | a |  |  | 0 |  |  |  |  |  |  |  | 1 |  |  |  |  |  |  |  |  |  |  |  |  |  |
|  | Flint rarity | Yes/No (a/b) | b |  |  | 0 |  |  |  |  |  |  |  | 1 |  |  |  |  |  |  |  |  |  |  |  |  |  |
|  | Flint quality | low/medium/high (a/b/c) | b |  |  |  |  |  |  | 0 | 0 |  |  | 1 |  |  |  |  |  |  |  |  |  |  |  |  |  |
| Action/Incisions | Agent | Knapped lithic/Grindstone/Bone,Antler,Wood (a/b/c) | a | 0 | 1 | 1 | 1 | 1 | 1 | 1 | 1 | 1 | 0 | 1 | 1 | 1 | 1 | 1 | 1 | 1 | 1 | 1 | 1 | 1 | 1 | 1 | 1 |
|  | Tool active area | linear/irregular/pointed (a/b/c) | c |  | 0 |  |  |  |  |  |  | 0 |  |  | 1 |  |  |  |  |  |  |  |  | 0 | 1 |  |  |
|  | Motion | single/back-and-forth (a/b) | a | 1 |  | 1 |  | 1 | 1 |  |  |  |  |  |  |  |  |  |  |  |  |  | 1 |  |  |  |  |
|  | Direction relative to one another | random/multiple/unique (a/b/c) | c | 0 | 0 |  |  | 1 | 1 |  |  |  | 0 | 1 | 1 |  | 1 |  |  |  | 1 |  | 1 |  | 1 |  | 1 |
|  | Direction relative to the cortex | parallel/oblique/perpendicular (a/b/c) | b |  |  |  |  |  |  |  |  |  |  |  |  |  |  |  |  |  |  |  |  |  |  |  |  |
|  | Direction relative to the object | parallel/oblique/perpendicular (a/b/c) | b |  | 0 |  |  | 0 |  |  |  |  |  |  |  |  |  |  |  |  | 0 |  |  |  |  |  |  |
|  | Depth of incisions | Shallow/moderate/deep (a/b/c) | b/c | 0 |  |  | 0.5 |  |  | 1 | 0 | 0.5 | 0 |  | 1 | 0.5 | 1 | 1 | 0.5 | 0 | 1 | 0.5 |  | 0.5 | 1 | 0.5 | 1 |
|  | Size of pattern relative to object size | Small/moderate/large (a/b/c) | b/c |  |  | 0.5 | 1 |  | 0.5 |  | 1 |  |  | 0 | 1 |  | 1 | 0.5 | 1 |  |  |  | 0.5 |  | 1 |  | 1 |
|  | Pattern consistency | low/medium/high (a/b/c) | b/c | 0 | 0 | 0 | 0 | 0 | 0 | 0 | 0 | 0 | 0 |  | 1 |  | 1 | 0 | 0 | 0 | 0 | 0 | 0 | 0 | 1 |  | 1 |
|  | Position | pervasive/eccentric/central (a/b/c) | a | 1 | 1 | 0 | 1 |  | 0 | 1 | 1 |  |  | 0 | 0 | 1 | 0 | 0 | 1 |  | 0 | 0 | 0 | 1 | 0 | 1 | 0 |
|  | Tool | single/multiple (a/b) | a | 1 | 1 | 1 |  |  |  | 1 | 1 |  |  |  |  | 1 |  |  |  |  |  |  |  |  |  | 1 |  |
|  | Number of incisions | low/medium/high (a/b/c) | c | 0 |  | 0 | 1 |  |  | 1 | 1 |  |  | 0 | 1 | 1 | 1 | 1 | 1 | 0 | 0 | 1 |  |  | 1 | 1 | 1 |
|  | Degree of neuromotor control | low/medium/high (a/b/c) | b/c | 0 | 0 | 0 | 0 | 0 | 0 | 0 | 0 | 0 | 0 |  | 1 |  | 1 | 0 | 0 | 0 | 1 |  | 0 | 0 | 1 |  | 1 |
|  | Traces of adhesive | Yes/No (a/b) | b |  |  |  |  |  |  |  |  |  |  |  |  |  |  |  |  |  | 0 |  |  |  |  |  |  |
| The probability score | | | | 0.27 | 0.30 | 0.41 | 0.61 | 0.33 | 0.39 | 0.73 | 0.64 | 0.44 | 0.00 | 0.63 | 0.89 | 0.79 | 0.88 | 0.64 | 0.61 | 0.50 | 0.44 | 0.58 | 0.39 | 0.45 | 0.89 | 0.79 | 0.88 |
